# Supplementary material for: From sequence to enzyme mechanism using multi-label machine learning
Source: BMC Bioinformatics. 2014 May 19;15:150. doi: 10.1186/1471-2105-15-150 (PMC4229970; doi:10.1186/1471-2105-15-150)
Supplement: Additional file 2 — Java code of ml2db. Additional file ml2db_code.tar.gz contains the Java source code to run the multi-label machine learning experiments and save the results to database. The code’s Javadoc is included. [file 1471-2105-15-150-S2.zip › additional file 2/ml2db/ml2db/doc/main/ml2db/al/utility/pickers/RandomPicker.html]

RandomPicker


JavaScript is disabled on your browser.


- Overview
- Package
- Class
- Use
- Tree
- Deprecated
- Index
- Help

- Prev Class
- Next Class

- Frames
- No Frames

- All Classes

- Summary:
- Nested |
- Field |
- Constr |
- Method

- Detail:
- Field |
- Constr |
- Method


main.ml2db.al.utility.pickers

## Class RandomPicker

- java.lang.Object
- - main.ml2db.al.utility.UtilityMetric
  - - main.ml2db.al.utility.pickers.RandomPicker

- All Implemented Interfaces:
  :   Picker

  Direct Known Subclasses:
  :   ByFrequencyPicker, LabelRandomPicker, RandomAttributePicker, RandomAttributeSetPicker, RandomInstancePicker, RandomLabelsPicker, RandomNeigbourhoodPicker

  ---

    

  ```
  public abstract class RandomPicker
  extends UtilityMetric
  implements Picker
  ```

  Class

  Version:
  :   25 Apr 2012

  Author:
  :   Luna De Ferrari ldf(at)st-andrews.ac.uk or
      luna.deferrari@st-andrews.ac.uk

- - ### Field Summary

    Fields

    | Modifier and Type | Field and Description |
    | --- | --- |
    | `boolean` | `m_debug` |
    | `boolean` | `m_test` |
  - ### Constructor Summary

    Constructors

    Constructor and Description

    `RandomPicker()`
  - ### Method Summary

    Methods

    | Modifier and Type | Method and Description |
    | --- | --- |
    | `uk.ac.standrews.utils.main.maputils.OneToManyMap<java.lang.String,java.lang.String>` | `getInstanceToFeaturesMap()` |
    | `abstract java.lang.String` | `getMetricName()` |
    | `java.util.Vector<IdInstance>` | `getQueries()` |
    | `IdInstance` | `getQuery(IdMultiLabelInstances unlabelled)` |
    | `int` | `getStep()` |
    | `double` | `getUtility(mulan.classifier.MultiLabelLearner trainedLearner, IdInstance instance)` |
    | `boolean` | `isDebug()` |
    | `boolean` | `isTest()` |
    | `void` | `refresh(IdMultiLabelInstances labelled, IdMultiLabelInstances unlabelled, IdInstance queryInstance)` Refresh the set of labelled and unlabelled instances |
    | `void` | `setDebug(boolean debug)` |
    | `void` | `setFeatureToInstanceMap(uk.ac.standrews.utils.main.maputils.OneToManyMap<java.lang.String,java.lang.String> featureToInstanceMap)` |
    | `void` | `setStep(int step)` |
    | `void` | `setTest(boolean test)` |

    - ### Methods inherited from class main.ml2db.al.utility.UtilityMetric

      `getUnlabelled, initialise, setUnlabelled`
    - ### Methods inherited from class java.lang.Object

      `equals, getClass, hashCode, notify, notifyAll, toString, wait, wait, wait`

- - ### Field Detail


    - #### m\_debug

      ```
      public boolean m_debug
      ```


    - #### m\_test

      ```
      public boolean m_test
      ```
  - ### Constructor Detail


    - #### RandomPicker

      ```
      public RandomPicker()
      ```
  - ### Method Detail


    - #### getInstanceToFeaturesMap

      ```
      public uk.ac.standrews.utils.main.maputils.OneToManyMap<java.lang.String,java.lang.String> getInstanceToFeaturesMap()
      ```


    - #### getMetricName

      ```
      public abstract java.lang.String getMetricName()
      ```

      **Specified by:**
      :   `getMetricName` in class `UtilityMetric`


    - #### getQueries

      ```
      public java.util.Vector<IdInstance> getQueries()
      ```

      Returns:
      :   m\_queries


    - #### getQuery

      ```
      public IdInstance getQuery(IdMultiLabelInstances unlabelled)
      ```


    - #### getStep

      ```
      public int getStep()
      ```

      Returns:
      :   m\_step


    - #### getUtility

      ```
      public double getUtility(mulan.classifier.MultiLabelLearner trainedLearner,
                      IdInstance instance)
      ```

      **Specified by:**
      :   `getUtility` in class `UtilityMetric`


    - #### isDebug

      ```
      public boolean isDebug()
      ```

      Returns:
      :   m\_debug


    - #### isTest

      ```
      public boolean isTest()
      ```

      Returns:
      :   m\_test


    - #### refresh

      ```
      public void refresh(IdMultiLabelInstances labelled,
                 IdMultiLabelInstances unlabelled,
                 IdInstance queryInstance)
      ```

      **Description copied from class: `UtilityMetric`**

      Refresh the set of labelled and unlabelled instances

      **Specified by:**
      :   `refresh` in class `UtilityMetric`

      Parameters:
      :   `unlabelled` - protected abstract void prepareQueryInstancesList(
          IdMultiLabelInstances unlabelled);


    - #### setDebug

      ```
      public void setDebug(boolean debug)
      ```

      Parameters:
      :   `debug` - m\_debug to set


    - #### setFeatureToInstanceMap

      ```
      public void setFeatureToInstanceMap(uk.ac.standrews.utils.main.maputils.OneToManyMap<java.lang.String,java.lang.String> featureToInstanceMap)
      ```

      Parameters:
      :   `featureToInstanceMap` - m\_featureToInstanceMap to set


    - #### setStep

      ```
      public void setStep(int step)
      ```

      Parameters:
      :   `step` - the active learning step


    - #### setTest

      ```
      public void setTest(boolean test)
      ```

      Parameters:
      :   `test` - m\_test to set


- Overview
- Package
- Class
- Use
- Tree
- Deprecated
- Index
- Help

- Prev Class
- Next Class

- Frames
- No Frames

- All Classes

- Summary:
- Nested |
- Field |
- Constr |
- Method

- Detail:
- Field |
- Constr |
- Method
